# Early Postoperative Cognitive Performance After Total Arch Replacement: A Prospective Observational Study

**DOI:** 10.1155/anrp/4717530

**Published:** 2026-08-10

**Authors:** Kyosuke Takahashi, Misa Kajitani, Wataru Takayama

**Affiliations:** ^1^ Department of Anesthesiology, Institute of Science Tokyo Hospital, Tokyo, Japan; ^2^ Department of Anesthesiology and Critical Care Medicine, Kawasaki Municipal Hospital, Kawasaki, Japan, kawasaki.jp; ^3^ Department of Anesthesiology, Kawasaki Saiwai Hospital, Kawasaki, Japan

**Keywords:** cardiopulmonary bypass, cognitive recovery, postoperative cognitive impairment, postoperative quality of recovery scale, total arch replacement

## Abstract

**Background:**

Early postoperative cognitive trajectories following total arch replacement remain insufficiently characterized. This study evaluated serial postoperative recovery patterns with particular focus on cognitive performance during the first postoperative week.

**Methods:**

Adult patients undergoing elective total arch replacement were enrolled. Recovery was assessed using the Postoperative Quality of Recovery Scale (PostopQRS) at baseline within 14 days before surgery and postoperatively at 3 h and 1, 3, and 7 days after extubation. Cognitive recovery was evaluated according to the revised PostopQRS cognitive recovery algorithm. Recovery trajectories including other PostopQRS domains were summarized descriptively.

**Results:**

Forty‐one patients were included in the final analysis. The mean (SD) age was 68.4 (10.4) years, and 31 patients (91%) were male. Cognitive recovery was observed in 23/41 (56.1%) of patients at 3 h after extubation, 26/41 (63.4%) at postoperative Day 1, 31/41 (75.6%) at postoperative Day 3, and 30/41 (73.2%) at postoperative Day 7. While physiological, emotive, and activities of daily living domains showed progressive improvement over time, nociceptive recovery remained incomplete throughout follow‐up and was primarily driven by persistent pain rather than nausea.

**Conclusion:**

Cognitive recovery after total arch replacement improved gradually during the early postoperative period but remained incomplete in a substantial proportion of patients at postoperative Day 7. These findings highlight the heterogeneous nature of postoperative cognitive recovery and support the importance of serial cognitive assessment after complex aortic surgery.

## 1. Introduction

Recovery after cardiovascular surgery involving cardiopulmonary bypass (CPB) is complex. In addition to procedure‐related complications, systemic inflammation, embolic phenomena, and hypoperfusion may contribute to adverse neurological outcomes. Neurological complications, including stroke and postoperative cognitive impairment, can substantially affect quality of life after surgery.

Surgical treatment for thoracic aortic disease is associated with a substantial risk of neurological complications because of prolonged CPB, hypothermic circulatory arrest, and manipulation of the aortic arch [1]. Although cerebral protection strategies such as antegrade selective cerebral perfusion under hypothermia have become standard practice [2], postoperative cognitive changes remain common. Prior studies have reported cognitive decline in approximately 40% of the patients several weeks after cardiac or aortic surgery [1, 3–5].

Importantly, cognitive performance after cardiac surgery may fluctuate over time. While some patients experience early impairment, many recover within months [6], suggesting that postoperative cognitive changes may be transient or dynamic rather than uniformly progressive. Early postoperative cognitive recovery is clinically meaningful for rehabilitation, discharge planning, and patient counseling. However, most existing studies have assessed cognitive outcomes at isolated postoperative time points, typically weeks to months after surgery [7]. Data describing serial recovery trajectories during the immediate postoperative period are limited, particularly after total arch replacement.

Therefore, the primary objective of this study was to characterize the early postoperative trajectory of cognitive recovery after total arch replacement using serial Postoperative Quality of Recovery Scale (PostopQRS) assessments. A secondary objective was to describe recovery patterns in the other PostopQRS domains.

## 2. Methods

This prospective observational study was conducted at a high‐volume cardiovascular surgery center from June 2016 to December 2016. The institutional review board approved the study protocol (approval ID: 28‐8). The study was conducted in accordance with the Declaration of Helsinki, and written informed consent was obtained from the participants. This study was conducted and reported in accordance with the Strengthening the Reporting of Observational Studies in Epidemiology (STROBE) statement.

Adult patients aged ≥ 18 years undergoing elective total arch replacement for thoracic aortic disease, including aortic aneurysm and aortic dissection, were eligible. Exclusion criteria were prior thoracotomy, psychiatric disorders, and preoperative cognitive impairment, defined as a Mini‐Mental State Examination (MMSE) score ≤ 21 [8].

Anesthesia was induced with propofol, rocuronium, and fentanyl and maintained with sevoflurane and remifentanil according to institutional practice. Total arch replacement was performed under moderate hypothermic circulatory arrest with antegrade selective cerebral perfusion during CPB. Core temperature was monitored using a bladder temperature probe, and the target temperature during circulatory arrest was approximately 26°C. Postoperatively, patients were extubated on the day after surgery provided there were no signs of surgical bleeding, hypoxemia, or altered level of consciousness.

Postoperative recovery was evaluated using the Japanese‐language version of the PostopQRS, which has been validated in Japanese surgical patients and assesses five domains: physiological, nociceptive, emotive, activities of daily living (ADL), and cognitive [9, 10]. The cognitive domain includes orientation, digits forward, and digits backward tasks assessing memory and attention.

Assessments were performed by trained physiotherapists within 14 days prior to surgery (baseline) and repeated postoperatively at 3 h and 1, 3, and 7 days after extubation. Physiotherapists performing PostopQRS assessments received training before study initiation. Formal blinding was not performed. Extubation was used as the postoperative reference time point, as anesthetic agents were administered continuously during mechanical ventilation. Recovery was defined according to PostopQRS recovery criteria. For the physiological, nociceptive, emotive, and ADL domains, recovery was defined as performance equal to or better than the individual baseline score. Cognitive recovery was evaluated using the revised PostopQRS cognitive recovery algorithm. The primary outcome was the trajectory of early postoperative cognitive performance over time. Secondary outcomes included postoperative trends in the other PostopQRS domains.

### 2.1. Statistical Analysis

Given the exploratory design of this study and the absence of prior data describing early cognitive trajectories after total arch replacement, a formal power calculation was not performed. The study aimed to enroll all eligible patients during the study period to provide preliminary estimates of recovery trajectories.

Patient characteristics were summarized using mean (standard deviation) or median [interquartile range] for continuous variables and counts with percentages for categorical variables.

The primary analysis focused on cognitive recovery according to the revised PostopQRS cognitive recovery algorithm. Cognitive recovery was defined using item‐level comparison with each patient’s baseline performance, incorporating published tolerance factors for normal variability in cognitive testing and adjustment for low baseline cognitive scores [11, 12].

The proportion of patients meeting cognitive recovery criteria was calculated at each postoperative timepoint. Recovery in the physiological, nociceptive, emotive, and ADL domains was evaluated as secondary outcomes. Recovery proportions for each domain and postoperative time point were summarized descriptively using counts and percentages. Recovery trajectories across PostopQRS domains and the pain and nausea subdomains of the nociceptive domain were summarized descriptively. Because some PostopQRS domains were not assessable in all patients during the immediate postoperative period, the number of evaluable patients differed across domains and time points.

Because of the modest sample size and exploratory nature of the study, no formal statistical comparisons across postoperative time points were performed. All analyses were performed using R Version 4.4.2 (R Foundation for Statistical Computing, Vienna, Austria).

### 2.2. Use of Artificial Intelligence (AI) Tools

No AI tools were used to generate the scientific content of this manuscript. AI‐assisted tools (e.g., ChatGPT and OpenAI) were used solely for language editing and formatting, and all content was reviewed and approved by the authors.

## 3. Results

**FIGURE 1:**
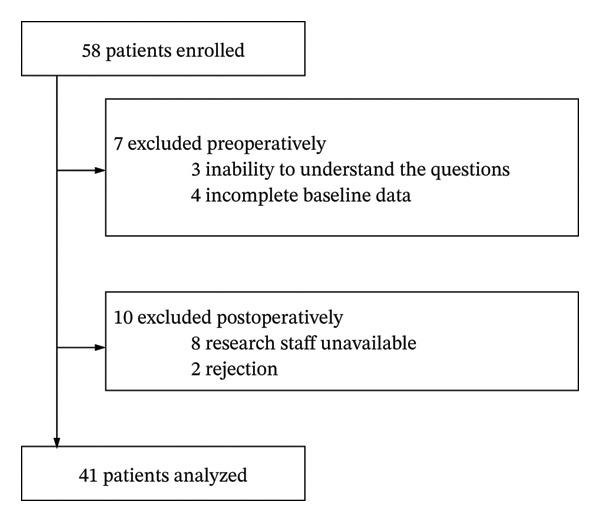
Flowchart of patients enrolled in the study.

During the study period, 58 patients were enrolled. Seven patients were excluded preoperatively because of inability to understand the questions or incomplete baseline assessments, and 10 were excluded postoperatively because of research staff unavailability or withdrawal of consent. Consequently, 41 patients were included in the final analysis (Figure 1).

Baseline characteristics are presented in Table 1. The mean (SD) age was 68.4 (10.4) years, and 31 patients (91%) were male. The median [IQR] duration of anesthesia was 454 [419–531] min, and the median [IQR] duration of CPB was 247 [198–273] min.

**TABLE 1:** Patient characteristics.

	Overall (*n* = 41)
Age, year, mean (SD)	68.8 (10.3)
Male sex, *n* (%)	36 (87.8)
Height, cm, mean (SD)	165.4 (9.0)
Weight, kg, mean (SD)	67.5 (17.8)
ASA PS, *n* (%)	
1	2 (4.9)
2	33 (80.5)
3	6 (14.6)
Smoking, *n* (%)	
Current	9 (22.0)
Ex	22 (53.7)
None	10 (24.4)
Cardiac disease, *n* (%)	11 (26.8)
Respiratory disease, *n* (%)	4 (9.8)
Education, *n* (%)	
Junior high school	3 (7.3)
High school	16 (39.0)
University	9 (22.0)
Post graduate	4 (9.8)
Unknown	9 (22.0)
Employment, *n* (%)	
Currently working	7 (17.1)
On leave due to the disease	8 (19.5)
Retired	17 (46.3)
Unknown	7 (17.1)
Baseline PostopQRS, median [IQR]	
Physiological	27 [27, 27]
Nociceptive	2 [2, 2]
Emotional	4 [3, 6]
Activity of daily living	4 [4, 4]
Cognitive	21 [19, 22]

*Note:* Data are presented as mean (SD), median [IQR], or *n* (%); PostopQRS, Postoperative Quality of Recovery Scale.

Abbreviation: ASA PS, American Society of Anesthesiologists Physical Status.

**FIGURE 2:**
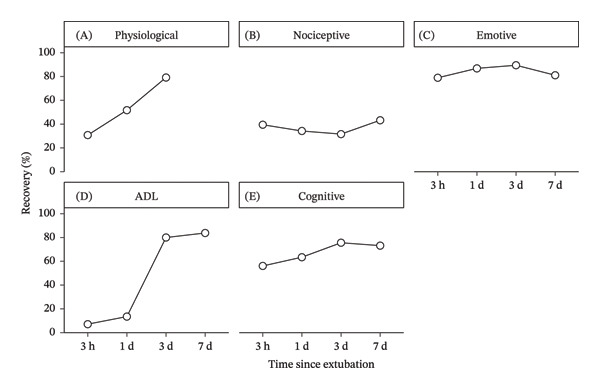
Recovery trajectories across postoperative quality of recovery scale domains. Proportions of patients meeting recovery criteria in the (A) physiological, (B) nociceptive, (C) emotive, (D) activities of daily living (ADL), and (E) cognitive domains at each postoperative time point. Data are presented as proportions (%). Recovery was defined according to the PostopQRS recovery criteria. For the physiological, nociceptive, emotive, and activities of daily living domains, recovery was defined as performance equal to or better than the individual preoperative baseline score. Cognitive recovery was determined using the revised PostopQRS cognitive recovery algorithm incorporating published tolerance factors and adjustment for low baseline cognitive scores.

Recovery patterns across the PostopQRS domains are summarized in Figure 2. Cognitive recovery improved gradually during the early postoperative period, with recovery observed in 23/41 (56.1%) of patients at 3 h after extubation, 26/41 (63.4%) at postoperative Day 1, and 31/41 (75.6%) at postoperative Day 3. At postoperative Day 7, cognitive recovery was observed in 30/41 (73.2%) of patients. Cognitive recovery improved gradually during the early postoperative period but remained heterogeneous across postoperative time points.

**FIGURE 3:**
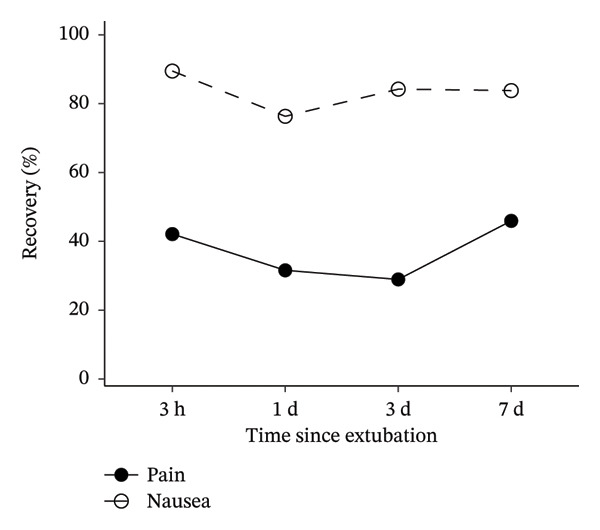
Recovery trajectories for the pain and nausea subdomains of the nociceptive domain. Proportions of patients meeting recovery criteria for pain and nausea at each postoperative time point. Recovery was defined as postoperative performance equal to or better than the individual preoperative baseline score.

Physiological recovery improved progressively during the early postoperative period, increasing from 8/26 (30.8%) at 3 h after extubation to 19/24 (79.2%) at postoperative Day 3. Nociceptive recovery remained incomplete throughout the observation period, ranging from 31.6% to 43.2% across postoperative assessments. Subdomain analysis demonstrated that recovery from nausea remained consistently high (76.3%–89.5%), whereas recovery from pain was substantially lower throughout the postoperative period (28.9%–45.9%) (Figure 3).

Emotive recovery was comparatively rapid, with 30/38 (78.9%) of the patients meeting recovery criteria at 3 h and 34/38 (89.5%) at postoperative Day 3. Recovery of ADL was limited during the immediate postoperative period but improved substantially over time, increasing from 1/14 (7.1%) at 3 h to 31/37 (83.8%) at postoperative Day 7.

## 4. Discussion

In this prospective observational study, recovery trajectories differed among PostopQRS domains during the first postoperative week after total arch replacement. While physiological, emotive, and ADL domains generally demonstrated progressive recovery over time, cognitive recovery remained incomplete in a substantial proportion of patients and showed considerable interindividual variability in recovery trajectories across postoperative assessments.

Most prior investigations of postoperative cognitive changes after cardiac surgery have evaluated outcomes at isolated time points, often weeks after surgery [7]. Reported rates of early postoperative cognitive impairment vary from 40% to 80% [3–5, 13, 14], likely reflecting differences in study design, assessment tools, and timing of evaluation. In our cohort, the proportion of patients meeting cognitive recovery criteria varied substantially across time points. These findings suggest that cognitive recovery during the early postoperative period is heterogeneous and may not be fully characterized by a single postoperative assessment.

The incomplete cognitive recovery observed in this study likely reflects the unique neurological challenges associated with total arch replacement. Although contemporary cerebral protection strategies, including hypothermic circulatory arrest and selective antegrade cerebral perfusion, have substantially reduced the incidence of overt neurological injury [15], subtle cognitive dysfunction may still occur [16]. Systemic inflammation, cerebral microemboli, hypoperfusion, and perioperative metabolic stress have been proposed as contributing factors to postoperative cognitive changes [17]. The considerable interindividual variability observed in our cohort further suggests that susceptibility to these perioperative insults may differ according to patient‐specific factors such as age, cognitive reserve, and cerebrovascular comorbidity. Further investigation incorporating biological or neuroimaging markers would be required to clarify underlying mechanisms.

These findings also have implications for perioperative neuroprotection strategies. Prevention of postoperative cognitive deterioration after complex aortic surgery likely requires a multidisciplinary approach spanning the preoperative, intraoperative, and postoperative phases [2, 17, 18]. Preoperative optimization, including physical and cognitive prehabilitation, may enhance physiological reserve before major surgery. Intraoperatively, strategies aimed at reducing cerebral embolism, maintaining adequate cerebral perfusion, and minimizing systemic inflammatory responses may mitigate neurological injury [2, 17]. Postoperatively, early mobilization, delirium prevention, and structured rehabilitation may help support recovery [18]. Although our study focused on early postoperative trajectories, future studies incorporating longer‐term follow‐up are needed to determine whether early incomplete cognitive recovery predicts persistent cognitive deficits [19].

In contrast, emotive recovery was rapid and sustained and ADL function showed steady improvement over time. However, the nociceptive domain demonstrated the lowest recovery rates, and exploratory analysis suggested that persistent pain rather than nausea appeared to account for most of the incomplete nociceptive recovery. Previous studies have suggested that greater postoperative pain intensity is associated with poorer postoperative cognitive outcomes [20] although the underlying mechanisms remain incompletely understood. These findings suggest that improved postoperative pain control may facilitate cognitive recovery after total arch replacement, a hypothesis that warrants further investigation. Taken together, domain‐specific differences underscore the multidimensional nature of postoperative recovery.

Clinically, our findings suggest that early postoperative cognitive improvement should be interpreted cautiously. Serial assessment may be more informative than single time‐point evaluation when planning rehabilitation intensity and discharge timing after total arch replacement.

Several limitations should be acknowledged. First, this was a single‐center study with a modest sample size, limiting generalizability. Second, although patients unable to complete cognitive assessments because of delirium, unconsciousness, or ongoing intubation were classified as nonrecovered according to revised PostopQRS methodology, some PostopQRS domains were not assessable in all patients at all timepoints. Third, follow‐up was limited to the first postoperative week; therefore, long‐term cognitive outcomes cannot be inferred. Finally, although the PostopQRS is a validated instrument, it is not equivalent to comprehensive neuropsychological testing and may not detect subtle deficits. Nevertheless, its feasibility for repeated bedside assessment enabled detailed characterization of early recovery patterns.

## 5. Conclusions

Cognitive recovery after total arch replacement improved gradually during the first postoperative week but remained incomplete in a substantial proportion of patients. Considerable interindividual variability was observed, highlighting the importance of serial cognitive assessment after complex aortic surgery. Further studies are warranted to clarify determinants of early cognitive instability and to develop strategies to optimize postoperative neurocognitive recovery.

## Funding

This research received no specific grant from any funding agency in the public, commercial, or not‐for‐profit sectors.

## Conflicts of Interest

The authors declare no conflicts of interest.

## Supporting Information

Additional supporting information can be found online in the Supporting Information section.

## Supporting information


**Supporting Information** STROBE Statement—Checklist of items that should be included in reports of observational studies.

## Data Availability

The deidentified data that support the findings of this study are available from the corresponding author upon reasonable request.
