# Supplementary material for: Early Postoperative Cognitive Performance After Total Arch Replacement: A Prospective Observational Study
Source: Anesthesiol Res Pract. 2026 Aug 10;2026:4717530. doi: 10.1155/anrp/4717530 (PMC13454786; doi:10.1155/anrp/4717530)
Supplement: Supplementary file 1 [file ANRP-2026-4717530-s001.pdf]

STROBE Statement—Checklist of items that should be included in reports of observational studies

Item No.	Recommendation	Location (line numbers)
**Title and Abstract**		
1a	Study design indicated in title/abstract	Title; Lines 33–36
1b	Informative and balanced abstract	Lines 33–57
**Introduction**		
2	Background/rationale	Lines 58–76
3	Objectives	Lines 79–81
**Methods**		
4	Study design	Lines 79–82
5	Setting	Lines 79–82
6	Participants	Lines 83–88
7	Variables (outcomes, exposures)	Lines 101–107
8	Data sources/measurement	Lines 101–108
9	Bias	Lines 106–109 (lack of blinding described)
10	Study size	Lines 111–114
11	Quantitative variables	Lines 115–116
12a	Statistical methods	Lines 117–134
12b	Subgroups/interactions	Not performed
12c	Missing data handling	Lines 131–134
12d	Sensitivity analyses	Not performed
**Results**		
13a	Participants at each stage	Lines 136–139
13b	Reasons for non-participation	Lines 136–138
13c	Flow diagram	Figure 1
14a	Descriptive data	Lines 138–141; Table 1
14b	Missing data	Line 150
15	Outcome data	Lines 141–152
16a	Main results	Lines 141–152
16b	Estimates and precision	Lines 142–145
16c	Absolute risk	Not applicable
17	Other analyses	Lines 146–152
**Discussion**		
18	Key results	Lines 153–158
19	Limitations	Lines 205–213
20	Interpretation	Lines 158–204
21	Generalizability	Lines 205–208

Item No.	Recommendation	Location (line numbers)
**Other Information**		
22	Funding	Lines 27–31
